# Redox-Related Neuronal Death and Crosstalk as Drug Targets: Focus on Epilepsy

**DOI:** 10.3389/fnins.2019.00512

**Published:** 2019-05-22

**Authors:** Xiao-Yuan Mao, Hong-Hao Zhou, Wei-Lin Jin

**Affiliations:** ^1^Department of Clinical Pharmacology, Xiangya Hospital, Central South University, Changsha, China; ^2^Institute of Clinical Pharmacology, Central South University, Hunan Key Laboratory of Pharmacogenetics, Changsha, China; ^3^National Clinical Research Center for Geriatric Disorders, Xiangya Hospital, Central South University, Changsha, China; ^4^Center for Translational Medicine, Ruikang Hospital, Guangxi University of Chinese Medicine, Nanning, China; ^5^Institute of Nano Biomedicine and Engineering, Department of Instrument Science and Engineering, Key Laboratory for Thin Film and Microfabrication Technology of Ministry of Education, School of Electronic Information and Electronic Engineering, Shanghai Jiao Tong University, Shanghai, China

**Keywords:** epilepsy, apoptosis, autophagy, necroptosis, pyroptosis, ferroptosis

## Abstract

Cell death has a vital role in embryonic development and organismal homeostasis. Biochemical, pharmacological, behavioral, and electrophysiological evidences support the idea that dysregulation of cell death programs are involved in neuropathological conditions like epilepsy. The brain is particularly vulnerable to oxidative damage due to higher oxygen consumption and lower endogenous antioxidant defense than other bodily organ. Thus, in this review, we focused on the comprehensive summarization of evidence for redox-associated cell death pathways including apoptosis, autophagy, necroptosis, and pyroptosis in epilepsy and the oxidative stress-related signaling in this process. We specially proposed that the molecular crosstalk of various redox-linked neuronal cell death modalities might occur in seizure onset and/or epileptic conditions according to the published data. Additionally, abundance of polyunsaturated fatty acids in neuronal membrane makes the brain susceptible to lipid peroxidation. This presumption was then formalized in the proposal that ferroptosis, a novel type of lipid reactive oxygen species (ROS)-dependent regulatory cell death, is likely to be a critical mechanism for the emergence of epileptic phenotype. Targeting ferroptosis process or combination treatment with multiple cell death pathway inhibitors may shed new light on the therapy of epilepsy.

## Introduction

**FIGURE 1:**
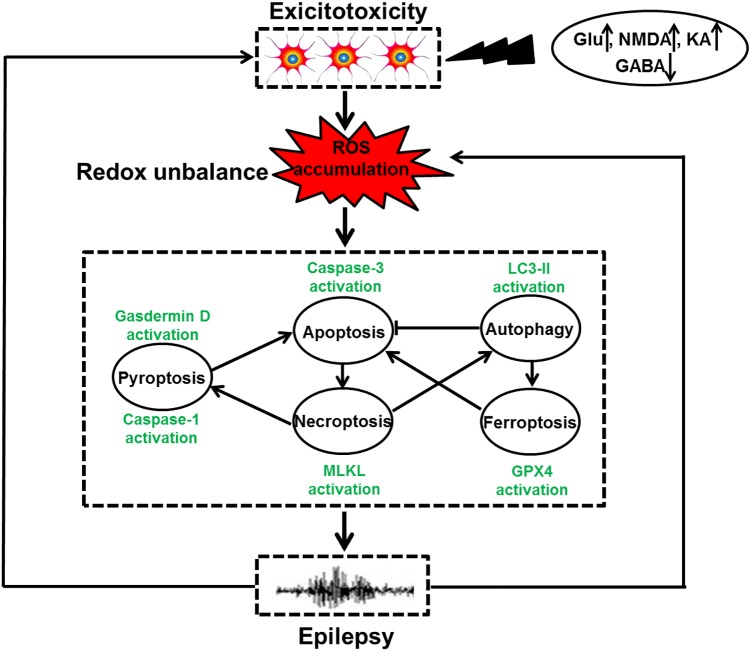
The relationship of redox-related cell death and epilepsy. Under the exposure of toxins, such as high levels of Glu, NMDA, and KA as well as low GABA content, neuronal hyperexcitation occurs, leading to ROS accumulation. Oxidative stress induced by redox unbalance then leads to multiple types of redox-associated neuronal death modes including apoptosis, autophagy, necroptosis, pyroptosis, and ferroptosis by activating caspase-3, LC3II expression, MLKL phosphorylation, caspase-1 activation and Gpx4 activity, respectively. Within a cell, the molecular crosstalk of these cell death modalities occurs, which ultimately triggers dead neuron phenotype and provokes epilepsy. Meanwhile, the recurrent epileptic seizures also have the capacity of exacerbating neuronal excitotoxicity and overproduction of ROS, shaping a vicious circle.

Cell death is a biological process that is essential for embryonic development and tissue homeostasis. Fine tuning of cell death in neurons is especially important due to the limited self-renewal capacity in adult neurons. Aberrant neuronal death is involved in multiple human diseases including epilepsy. The role of neuronal death mechanisms in epilepsy has been debated for decades. Despite the possibility that isolated brief seizure may not induce cell death, severe and repetitive seizures can undoubtedly activate neuronal death process (Dingledine et al., 2014). And seizure-evoked cell death pathway may have several detrimental effects. It is well known that repeated brief seizures lead to progressive hippocampal neuron loss and spatial memory deficits (Kotloski et al., 2002). Experimental data also indicate that rats with seizure-induced hippocampal damage show prolonged episodes of recurrent seizures and more frequent severe epileptic seizures (Zhang et al., 2002). Delineating the molecular mechanisms underlying seizure-induced neuronal death may obviate the deleterious impact of seizures on the brain and/or slow the progression of epilepsy. Currently, neuronal death modalities can be classified into accidental and regulated forms according to distinct morphological features. Accidental neuronal death often does not involve a specific molecular mechanism and these cells cannot be rescued. In contrast, regulated cell death involves the cellular machinery and this death mode can be manipulated by pharmacological and genetic ways (Galluzzi et al., 2015). With the progress in investigating the regulated cell death mechanisms, multiple paradigms of death modes including apoptosis, necroptosis, autophagy, pyroptosis, entosis, cornification, parthanatos, and ferroptosis have been identified on the basis of their respective executive mechanisms (Galluzzi et al., 2018). Biochemical, pharmacological, behavioral and electrophysiological evidences support that various factors have been reported to cause neuronal cell death. These may involve oxidative stress, inflammation, excitotoxicity and so on (Ferriero, 2005). The contribution of oxidative stress to neuronal death attracts considerable attention in epilepsy research for the following reasons. First, the brain is an organ that is highly susceptible to oxidative stress because it consumes the highest amounts of oxygen (approximately 20% of oxygen) than other bodily organ and the brain has weak anti-oxidant defense systems (Cobley et al., 2018). Second, there are abundant polyunsaturated fatty acids in neuronal membrane that are prone to lipid peroxidation, a central feature of oxidative stress (Cobley et al., 2018). Third, the brain is rich in iron and copper, which facilitate the catalysis of hydroxyl radical formation (Valko et al., 2007; Waldbaum and Patel, 2010). Neuronal cells in the brain are particularly vulnerable to oxidative damage. Under pathological situation, oxidative stress is induced due to excessive ROS caused by disturbed redox homeostasis and subsequently makes neurons succumb to distinct types of cell demise including apoptosis, autophagy, necroptosis, pyroptosis, and ferroptosis (Figure 1). Experimental evidence has also supported the idea that prolonged seizures invoke oxidative stress and consequently trigger neuronal death (Liang et al., 2000; Patel et al., 2001; Mao et al., 2018), indicating a key role of oxidative stress in seizure-induced neuronal death and/or the development of epilepsy. Our review article aimed to update basic knowledge of roles of various redox-associated neuronal death modes including apoptosis, autophagy, necroptosis, pyroptosis and ferroptosis as well as their interactions in epilepsy, provide our current understanding of the possible mechanism especially their regulation by redox, and describe the potential therapeutic interventions.

## Redox-Associated Apoptosis in Epilepsy

**FIGURE 2:**
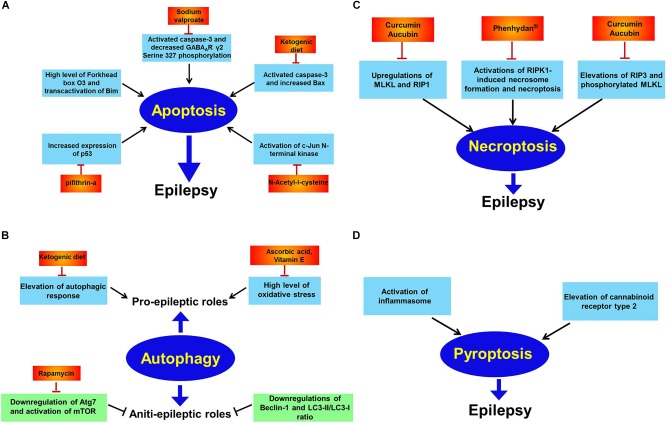
Apoptosis, autophagy, necroptosis, and pyroptosis in epilepsy. Panel **(A)** indicates apoptosis in epilepsy. Extensive investigations has illustrated elevations of oxidative stress, Forkhead box O3, p53, and c-Jun N-terminal kinase while decreased GABA_A_R γ2 Serine 327 phosphorylation are reported to trigger neuronal apoptosis and subsequently promote epileptic phenotype. And many drugs including sodium valproate, pifithrin-a, *N*-acetyl-l-cysteine and supplement with ketogenic diet all counteract epileptic seizures via inhibiting apoptosis; panel **(B)** shows autophagy in epilepsy. Autophagy has dual effects on epilepsy. That is pro-epileptic and anti-epileptic roles. On the one hand, induction of autophagic response by high level of oxidative stress has been found to cause epilepsy. Diverse therapeutic interventions including ascorbic acid, vitamin E and ketogenic diet exert curative potential against epilepsy. On the other hand, autophagic activation by rapamycin has therapeutic effect against epilepsy via inhibition of mTOR; panel **(C)** shows necroptosis in epilepsy. Activations of RIP1, RIP3, MLKL, and phosphorylated MLKL have been reported to induce necroptosis and contribute to epileptogenesis. However, curcumin, aucubin or phenhydan^®^ suppresses epileptic seizures via inhibiting necroptosis; panel **(D)** shows pyroptosis in epilepsy. Activation of inflammasome and elevation of cannabinoid receptor type 2 have been observed to result in pyroptosis in animal models of epilepsy.

Apoptosis, firstly identified in 1972, is a morphologically distinct type of cell death characterized by cell shrinkage, DNA fragmentation and chromatin condensation (Kerr et al., 1972). It is often evoked by two major pathways. In the extrinsic pathway, apoptotic cell death is induced by death receptors, whereas, in the intrinsic pathway, death signals directly or indirectly regulate mitochondria, leading to the release of cytochrome c and generation of the apoptosome complex (Fricker et al., 2018). Several human and experimental studies have revealed the relationship between neuronal apoptosis and epilepsy. Although whether apoptosis is a cause or consequence of epileptogenesis remains controversial, it has been widely demonstrated that activation of apoptotic signaling pathway can exacerbate seizure-induced brain impairment and cause prolonged epileptic seizures (Figure 2A) (Zhang et al., 2002). Mitochondria in the brain are prone to being disrupted during epileptic seizures. In response to seizure-induced brain insult, mitochondria permeabilization occurs and proapoptotic proteins (cytochrome C and AIF) are released from mitochondria, resulting in the activation of downstream executioner caspase (caspase-3) and neuronal apoptosis (Engel and Henshall, 2009). Additionally, as mitochondria are the predominant sites of ROS generation, they are particularly vulnerable to oxidant damage. It has supported that during epileptic seizures, neuronal excitotoxicity promotes the elevation of intracellular Ca^2+^ level in mitochondria, resulting in ROS overproduction (Brennan et al., 2009; Fricker et al., 2018). Ca^2+^-dependent mitochondrial ROS accumulation initiates the opening of mPTP, which permits the release of cytochrome C from the mitochondria to cytosol, triggering caspase-3-dependent apoptotic cell death. These data suggest that targeting mitochondrial oxidative stress and dysfunction may be a new strategy for blunting neuronal apoptosis, alleviating seizure occurrence and/or suppressing epileptogenesis. Mitochondria-independent oxidative stress can also exist in specific context as studies have shown that engagement of ligand-death receptor (e.g., Fas ligand–Fas receptor) in the extrinsic pathway of apoptosis causes lipid raft formation and Nox activation and ROS production (Finkel, 2003). In this way, a combination of these processes including ROS generation ultimately facilitates activation of death receptor and induction of apoptosis. This suggests that mitochondria-independent oxidative stress may constitute another mechanism that converges into neuronal apoptosis in seizures and/or epilepsy.

## Oxidative Stress Signaling and Neuronal Apoptosis

### Excitotoxicity

Neuronal excitotoxicity involves excessive excitatory neurotransmission that is primarily mediated by glutamate. A high concentration of extracellular glutamate results in activation of NMDA receptor which in turn allows entry of Ca^2+^ into the neuronal cells (Fricker et al., 2018). The subsequent accumulation of Ca^2+^ elicits ROS production which activates various cell death scenarios including apoptosis via opening of mPTP (Figure 1). Evidence for the involvement of neuronal excitotoxicity in seizure onset and/or epileptic conditions came from studies showing that the concentration of glutamate was extraordinarily elevated in epileptic animal models and human epilepsy (Lipton and Rosenberg, 1994; Li Q. et al., 2018). Inhibition of GABA_A_R-mediated inhibitory neurotransmission also contributes to neuronal hyperexcitation and causes apoptosis. In our previous investigation, it was found that suppression of GABA_A_R γ2 Serine 327 phosphorylation was sufficient for aggravating neuronal apoptosis in a mouse model of KA-induced epileptic seizures and this phenomenon was prevented by sodium valproate (Li Q. et al., 2018). Taken together, these results indicate that neuronal excitotoxicity serves as an important oxidative stress-responsive pathway and seizure-induced apoptosis in epilepsy.

### Forkhead Box O3 (FOXO3)

The FOXO subfamily FOXO3 is one of the best characterized transcription factors that generate ROS. It seems that FOXO3 and ROS are regulated with each other in neurons. On the one hand, FOXO3 results in uncoupling of mitochondrial respiration in a Bim-dependent way, which subsequently activates overproduction of ROS and neuronal apoptosis (Hagenbuchner et al., 2012). Despite the negative role of FOXO3 in ROS regulation has been found in bovine aortic endothelial cells (Olmos et al., 2013). It has revealed that FOXO3-mediated ROS accumulation may be, to some extent, associated with interference of various genes coding for antioxidant proteins such as thioredoxin and thioredoxin reductase in mitochondria. The differential roles of FOXO3 in ROS modulation may be linked with cellular context. On the other hand, in response to many apoptotic stimuli (e.g., etoposide or doxorubicin), increased level of ROS promotes the nuclear translocation of FOXO3 and transactivates the expression of Bim in the downstream (Hagenbuchner et al., 2012). In patients with temporal lobe epilepsy, high level of FOXO3a was found in the mitochondrial fraction of hippocampus (Caballero-Caballero et al., 2013), which indicates that FOXO3 may act as a target that affects neuronal apoptosis via modulation of ROS generation in brain pathologies including epilepsy.

### p53

In neurons, p53 is a pleiotropic transcription factor that can mediate apoptosis under exposure to a range of cellular stresses such as oxidative stress and hypoxia (Morrison and Kinoshita, 2000). The detailed mechanism of activation of p53 under oxidative stress conditions is poorly understood, but oxidative DNA damage serves as a possible reason for initiating p53-mediated neuronal apoptosis. It has demonstrated that the p53 inhibitor PFT cannot reduce oxidative stress, but significantly attenuate neuronal apoptosis via decreasing expression of p53 target gene *Bax* and suppressing mitochondrial dysfunction and caspase activation (Culmsee et al., 2001). This gives rise to the concept that p53 in neurons serves as an important regulator of ROS-mediated apoptotic signaling upstream of abnormal mitochondrial function. Evidence for a pivotal role of p53 in neuronal apoptosis in seizures or epilepsy is provided by data documenting increased p53 expression in animal models of epilepsy (Culmsee et al., 2001) and patients with temporal lobe epilepsy (Engel et al., 2007). Pharmacological inhibition of p53 by PFT significantly protects neurons from apoptosis in a model of KA-induced seizures (Culmsee et al., 2001). Although how p53 contributes to redox response remains confused in neuronal apoptosis, p53-mediated up-regulation of redox-related enzymes including manganese superoxide dismutase and glutathione peroxidase has been found in fibroblast (Hussain et al., 2004), suggesting that p53-dependent oxidative stress and subsequent apoptotic cell death are the result of an imbalance in antioxidant enzymes by p53. Overall, these findings expose p53 as a key factor associated with neuronal apoptosis and p53 inhibition may act as an attractive therapeutic approach to prevent seizure-induced brain damage in human epilepsy.

### Mitogen-Activated Protein Kinases (MAPKs)

It has depicted that ROS-mediated oxidative stress can activate the pathway of MAPKs including ERK, JNK, and p38 in neurons (Samanta et al., 1998; Kamata et al., 2005; Yang et al., 2018). The prevention of ROS accumulation by a ROS scavenger, namely, NAC, abrogates JNK and p38 MAPK activation and subsequently promotes neuronal survival under NMDA-induced excitotoxic conditions (Yang et al., 2018), indicating the critical role of ROS-mediated JNK and p38 MAPKs activation in neuronal apoptosis. Although the mechanism for ROS-mediated activations of JNK and p38 MAPK is not well understood, it implicates that JNK and p38 serve as the potential targets for modulating neuronal apoptosis during seizures and/or epileptogenesis. The results showing resistance to neuronal apoptosis and seizures in Jnk3 (one member of JNK family)-deficient mice (Yang et al., 1997) confirms the contribution of JNK activation to seizure-induced apoptotic cell death. In terms of ERK, whether it has a role in regulation of apoptosis in neurons remains controversial. Studies has shown that ERK blockade cannot suppress staurosporine- or TNFα-induced HT22 neuronal apoptosis (Satoh et al., 2000), indicating that ERK activation may preferentially trigger non-apoptotic cell death including necrosis. Our postulation is supported by another report on a cell model of seizure activity (Murray et al., 1998).

## Redox-Associated Autophagy in Epilepsy

Autophagy is a lysosomal degradation of excessive or defective macromolecules and organelles and has a crucial role for energy supply and molecular building blocks via reusing the macromolecule after the nutrient stimuli (Pasquali et al., 2009; Mizushima and Komatsu, 2011). It has shown that Autophagy is modulated by multiple factors, among which the rapamycin complex 1 (mTORC1), one of the functional complexes of mTOR, is considered as the best characterized repressor of autophagic responses (Laplante and Sabatini, 2012). Specially, the relationship between mTORC1 and autophagic response is more tightly linked by nutrient stress. For details, mTORC1 is activated and the autophagy is repressed by inhibiting Ulk1 complex in the presence of nutrients while mTORC1 is suppressed and subsequent initiate Ulk1 complex-mediated autophagosome formation in the shortage of nutrients (Jung et al., 2010; Kim and Guan, 2015). What’s more important, there is evidence showing that starvation induces ROS formation and subsequently activates autophagic responses (Scherz-Shouval et al., 2007), suggesting that ROS occurs upstream of autophagy. Currently, whether induction of autophagy has a beneficial or deleterious role is not well understood. Characteristics of autophagy have been observed in various types of epilepsy models. Elevations of LC3-II/LC3-I ratio and Beclin-1 expression have been found in PTZ-, KA- or pilocarpine-induced epilepsy (Figure 2B) (Dong et al., 2013; Zhu et al., 2016; Hussein et al., 2018; Wang L. et al., 2019). However, whether autophagy has a causal role in promoting seizure onset or epileptogenesis is still unclear. Accumulating data show that suppressed autophagy activity may contribute to epilepsy. The study by McMahon research group shows that disinhibition of mTOR by deleting upstream *Tsc1* or *Pten* gene in neurons impairs autophagy and evokes seizure generation in the genetic epilepsy, TSC and other types of epilepsy including acquired temporal lobe epilepsy, progressive myoclonus epilepsy, and absence seizures (Wong, 2010; Polajnar and Zerovnik, 2011; McMahon et al., 2012). At the same time, genetic inactivation of *Atg7*, an important promoter of autophagy, also leads to spontaneous seizures in mice (McMahon et al., 2012). These findings suggest that activation of autophagic response, using autophagy inducers, such as rapamycin, may be an exciting therapeutic approach for epilepsy treatment and/or prevention. On the other hand, activation of autophagy may also contribute to neuronal death in epileptic conditions, particularly in response to oxidative stress (Cao et al., 2009a,b). Suppression of oxidative stress via antioxidants vitamin E and ascorbic acid significantly inhibits autophagic response in pilocarpine-induced status epilepticus (Cao et al., 2009a,b), suggesting that the role of autophagy in epilepsy may be altered under oxidative stress conditions, although the regulatory mechanism of oxidative stress in autophagic process is unknown.

**Table 1:** Features of epilepsy consistent with ferroptosis.

Feature	Description	References
Elevated lipid peroxidation products	F2-Isoprostanes, 4-hydroxy-2-nonenal protein adducts and malonaldehyde were enhanced.	Pecorelli et al., 2015; Zhu et al., 2016
ACSL4 inhibitors reduced epileptic seizures	Treatment with pioglitazone, a recently discovered ACSL4 inhibitor, abrogated the increased risk of epilepsy.	Abdallah, 2010; Simeone et al., 2017
12/15-lipoxygenase inhibitors reduced the risk of epileptic seizures	Baicalein, a 12/15-lipoxygenase inhibitor, was found to abnegate the increased risk of epilepsy in rodents.	Mao et al., 2014
Increased 5-lipoxygenase activity	5-Lipoxygenase activity was augmented in KA-induced epilepsy.	Manev et al., 1998
GPX4	Selenocysteine-containing GPX4 prevented epileptic seizures.	Ingold et al., 2018
Iron overload	Accumulation of iron was found in epileptic patients; injection of iron in brain could trigger epileptic discharges.	Willmore et al., 1978a,b
Possible clinical benefits of iron chelation	Treatment with deferoxamine, a common iron chelator, could suppress epilepsy.	Zou et al., 2017


*The listed features of epileptic seizures were in line with a role for ferroptosis in the disease etiology.*

## Redox-Associated Necroptosis in Epilepsy

Necroptosis represents one of the best-characterized types of regulated necrosis in cells. This cell death mode is mediated by RIP3 protein and its substrate, MLKL protein (Vandenabeele et al., 2010). During necroptosis, RIP1 is activated which further results in the activation of RIP3, promoting MLKL phosphorylation and forming a complex called necrosome (Conrad et al., 2016). RIP3 activation recruits and phosphorylates MLKL to facilitate its oligomerization and translocation to the plasma membrane, leading to membrane rupture and necrosis (Conrad et al., 2016). It is widely accepted that the formation of necrosome and the phosphorylation of MLKL are primary events during necroptotic cell death and therefore regarded as the hallmarks of necroptosis (Vandenabeele et al., 2010). Recent investigations have shown that necrosome formation/activation results in mitochondria dysfunction by disturbing ROS, thus exerting an inducible effect on necroptotic cell death process. Mounting evidence validates the contribution of mitochondrial ROS accumulation to necroptosis *in vitro* (Kim et al., 2007; Ye et al., 2012). The reason why ROS is involved in necroptosis may come from the finding showing that MLKL phosphorylation at Thr357 and Ser358 leads to the overexpression of ROS and subsequent necrotic cell death (Shulga and Pastorino, 2012).

Necroptotic features including elevations of RIP1, RIP3, and MLKL and membrane rupture have been observed in status-epilepticus, supporting the idea that the occurrence of necroptosis in epilepsy (Cai et al., 2017). And suppression of necroptosis by chemical reagents (e.g., curcumin, aucubin, and phenhydan^®^) significantly protects neurons against seizure-induced hippocampal damage (Figure 2C) (Wang et al., 2017a,b; Moerke et al., 2018). As such, necroptotic cell death pathway may be a useful therapeutic target for brain insults induced by seizures and/or epilepsy. Despite unclear mechanism of necroptosis pathway in epileptic conditions, oxidative stress may act as a positive role in this process as recent investigations have revealed a contribution of ROS accumulation to necroptotic cell death in various cell models (Zhang et al., 2016; Zhao et al., 2017; Han et al., 2018). Further study is needed to clarify this fact in epilepsy research.

## Redox-Associated Pyroptosis in Epilepsy

Pyroptosis, which was coined in 2001, is a type of necrotic cell death that is activated by inflammatory caspases, particularly caspase-1 (Cookson and Brennan, 2001). During pyroptosis, caspase-1 cleaves and activates GSDMD and causes NLRP3 inflammasome formation, the primary feature of pyroptosis. Accumulating data supports the idea that, when activated, the pyroptotic death pathways can induce the release of various inflammatory effector molecules including IL-1β and IL-18 and subsequently possibly affect nearby cells, finally facilitating leukocyte entry into the brain due to blood-brain barrier breakdown (Fink and Cookson, 2005; Zou et al., 2017). The activated inflammasome has been reported in patients with intractable mesial temporal lobe epilepsy and knock down of caspase-1 can alleviate seizure-induced neuronal damage in amygdala kindling-induced rat model, indicating occurrence of pyroptosis in epilepsy (Figure 2D) (Tan et al., 2015). Similarly, neuronal pyroptosis has also been identified in pilocarpine-induced status epilepticus and cannabinoid receptor type 2 are found to be overtly increased in this process, indicating an inducible role of cannabinoid receptor type 2 in neuronal pyroptosis pathway (Figure 2D) (Taabazuing et al., 2017). Disrupted ROS generation is often associated with cell pyroptosis. It has shown that GSDMD cleavage is reduced by the elimination of ROS and ROS accumulation by H2O2 treatment enhances caspase-1-dependent GSDMD activation (Wang Y. et al., 2019). Wang et al. further found that the oxidation of cysteines on GSDMD protein was triggered under oxidative stress (Wang Y. et al., 2019). These data imply that ROS leads to inflammasome-dependent pyroptotic cell death via GSDMD oxidation. Further investigation is essential to clarify this mechanism in epilepsy.

## Is Redox-Associated Ferroptosis Important in Epilepsy?

Ferroptosis is an iron-dependent mode of regulated cell death that is characterized by the accumulation of lethal lipid-based ROS (Dixon et al., 2012; Li Q.-Q. et al., 2018). It has the unique morphological characteristics and involves genetic, metabolic and execution mechanisms that are for the most part not distinct from other forms of cell death events such as apoptosis, autophagy, necroptosis and so on. Morphologically, ferroptotic cells involve mitochondria that are smaller than normal with condensed membrane densities. Lipid ROS overproduction is another evident feature of ferroptotic cell death. Nowadays, ferroptosis process has been extensively reported in various diseases including cancers (Alvarez et al., 2017; Viswanathan et al., 2017), neurological disorders (Chen et al., 2015; Hambright et al., 2017) and renal failure (Friedmann Angeli et al., 2014). Strikingly, several ferroptotic regulators such as Gpx4 and ALOX5 have been involved in hippocampal neurodegeneration *in vivo* and in glutamate-induced neurotoxicity *in vitro*. It has demonstrated that inactivation of Gpx4 causes neuronal death in a pathological relevant form of ferroptosis and promotes cognitive deficits and neurodegeneration (Chen et al., 2015; Hambright et al., 2017). The investigation by Liu research group has disclosed an increased ALOX5 activity in ferroptosis process under glutamate-induced neurotoxicity and suppression of ALOX5 by zileuton abrogates ferroptosis in this model. Taken together, these data implicate that Gpx4 serves as an inhibitory factor while ALOX5 exerts a positive role in the execution of neuronal ferroptosis. In epileptic conditions, there are direct and indirect evidence showing occurrence of neuronal ferroptosis as we make a summarization in Table 1. The direct evidence arises from the study revealing that smaller mitochondria are found in rat hippocampus of KA-induced temporal lobe epilepsy (Ye et al., 2019). The literature is rife with examples of indirect ferroptotic signs including iron accumulation in various animal models of epilepsy. Biochemical evidence also supports the occurrence of ferroptosis in the etiology of epilepsy as lipid degradation products such as 4-HNE and MDA are found to be enormously augmented (Willmore et al., 1978a,b; Manev et al., 1998; Abdallah, 2010; Mao et al., 2014; Pecorelli et al., 2015; Zhu et al., 2016; Simeone et al., 2017; Zou et al., 2017). And reduction of iron content by iron chelation agent (e.g., deferoxamine) suppresses epileptic seizures (Zou et al., 2017). Although the ferroptosis inhibitor ferrostatin-1 was not previously found to alleviate epileptic seizures induced by KA, improvements in cognitive deficits were documented in this model, suggesting the therapeutic potential of ferroptosis inhibition in seizure-induced cognitive decline. Collectively, these findings suggest that ablation of neuronal ferroptosis is likely to be a novel therapeutic approach for epileptic patients and this strategy may be suitable for other patients who are refractory to the traditional epileptic drugs.

## Crosstalk of Redox-Associated Neuronal Death in Epilepsy

**FIGURE 3:**
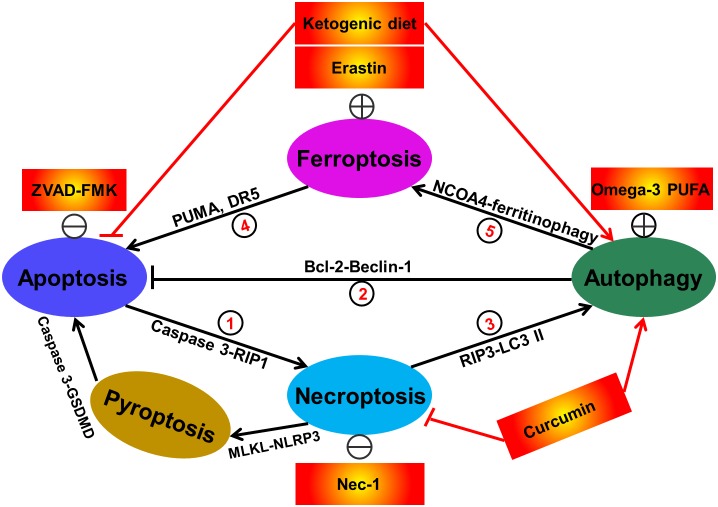
Interactions between different types of redox-related cell death including apoptosis, autophagy, necroptosis, and ferroptosis. Currently, at least seven sorts of crosstalk of redox-regulated cell death have existed in cells. In details, apoptosis induction by caspase-3 activation promotes RIP1-dependent necroptosis. This phenomenon can be reversed by treatment with caspase inhibitor ZVAD-FMK (①). Activation of autophagic response by increased Beclin-1 has the capacity of abrogating cellular apoptosis. The direct evidence supporting this fact is that administration of omega-3 polyunsaturated fatty acid has obviously attenuated neuronal apoptosis in a rat model of traumatic brain injury via inducing SIRT1-mediated deacetylation of Beclin-1 (②). Suppression of necroptosis has also been shown to activate autophagic flux. For example, treatment with the necroptosis inhibitor Nec-1 has been demonstrated to alleviate hepatocyte autophagy and subsequently suppress liver damage (③). Ferroptosis activation by erastin has been reported to trigger cell apoptosis via upregulating apoptosis-relating factors including PUMA and death receptor 5 (④). Additionally, autophagy has also been illustrated to be a positive regulator of ferroptotic cell death (⑤), due to the previous investigations showing that NCOA4 (nuclear receptor coactivator 4)-mediated degradation of ferritin and elevation of intracellular Fe^2+^ levels (namely ferritinophagy) contributes to ferroptosis in mouse embryonic fibroblasts. In some cases, some therapeutic interventions including ketogenic diet and curcumin are able to simultaneously regulate diverse cell death modes. The apoptotic executioner caspase-3 specially blocks GSDMD-mediated pyroptosis (⑥). The evidence for the contribution of necroptosis to pyroptosis arises from the fact showing that activated MLKL triggers NLRP3-mediated inflammasome formation (⑦).

Since the identification of various redox-related cell death modes, studies on the crosstalk between these death modes regulation have never stopped. The distinct modes of cell death are often modulated by similar signaling pathways, organelles, common subcellular sites and even manipulated by shared death initiator and effector molecules. In particular, the molecular crosstalk of cell death process is often induced by common cellular stressors such as energy/ATP levels and ROS (Amaravadi and Thompson, 2007; Scherz-Shouval et al., 2007; Cao et al., 2011). In some cases, ATP depletion results in autophagic response. Nevertheless, when autophagy fails to retain ATP level, necroptosis is triggered (Amaravadi and Thompson, 2007). Besides, as described above, ROS has the capacity of inducing apoptosis, autophagy, necroptosis, pyroptosis, and ferroptosis. ROS stimuli may simultaneously trigger multiple types of cell death within a cell. These cell death modalities can interact with each other, but ultimately one cell death mechanism dominates others. The interplay of the redox-associated cell death (including apoptosis, autophagy, necroptosis, pyroptosis and ferroptosis) is very complex. To our knowledge, until now, at least seven sorts of crosstalk of redox-regulated cell death have existed in cells as shown in the following (Figure 3): crosstalk between apoptosis and necroptosis; crosstalk between apoptosis and autophagy; crosstalk between autophagy and necroptosis; crosstalk with pyroptosis and apoptosis; crosstalk with pyroptosis and necroptosis; crosstalk between ferroptosis and apoptosis as well as crosstalk between ferroptosis and autophagy. Following hepatic ischemia-reperfusion injury, activation of apoptosis-related factor caspase-3 has been found to promote RIP1-dependent necroptosis (Rosentreter et al., 2015). And activated autophagic response may also abrogate apoptotic cell death pathway as a recent study has illustrated induction of SIRT1-mediated deacetylation of Beclin-1 (an important autophagic regulator) results in the suppression of neuronal apoptosis in a rat model of traumatic brain injury (Chen et al., 2018). In another research, suppression of necroptosis by the selective inhibitor, Nec-1, has obviously inhibited autophagic responses and attenuated hepatic ischemia (Hong et al., 2016). Similarly, the peroxisome proliferator-activated receptor-γ agonist troglitazone-induced autophagy in bladder cancer was partially blocked by Nec-1 and subsequently troglitazone-triggered loss of cell viability was rescued, indicating the existence of the interaction between autophagy and necroptosis (Yan et al., 2014). The crosstalk between apoptosis and pyroptosis has also occurred as a recent work has illustrated that the apoptotic executioner caspase-3 specially blocks GSDMD-mediated pyroptosis (Taabazuing et al., 2017). The evidence for the contribution of necroptosis to pyroptosis arises from the fact showing that activated MLKL triggers NLRP3-mediated inflammasome formation (Conos et al., 2017). In terms of ferroptosis, a recently discovered type of cell death, the crosstalk of it and other redox-associated cell death has also been extensively reported. Induction of ferroptosis has been shown to promote cell apoptosis in cancers. For instance, in human pancreatic cancer PANC-1 and BxPC-3 and colorectal cancer HCT116 cells, treatment with ferroptosis inducer erastin remarkably enhanced TRAIL-induced cell apoptosis via upregulating the expression of apoptosis regulator PUMA (Hong et al., 2017). Likewise, when treatment with ferroptotic agents such as erastin, sorafenib and artesunate in human colon cancer HCT116, CX-1, and LS174T cells, TRAIL-induced apoptosis was further augmented via upregulation of DR5 (Lee et al., 2019). Hou et al. (2016) revealed that knockout or knockdown of Atg5 and Atg7 evidently inhibited erastin-induced ferroptosis with decreased intracellular Fe^2+^ levels in fibroblasts and cancer cells. And in this process, NCOA4 was responsible for degradation of ferritin and elevation of intracellular Fe^2+^ levels (namely ferritinophagy) (Hou et al., 2016). It suggests that autophagy is a positive regulator of ferroptotic cell death. Similarly, it was also confirmed that NCOA4-mediated ferritinophagy also exacerbated cellular labile iron and ROS, leading to ferroptosis in mouse embryonic fibroblasts (Gao et al., 2016). Taken together, these findings imply that diverse types of cell death modalities are likely to co-exist and interact with each other in the pathological conditions including epilepsy and targeting one type of cell death may not obtain satisfactory effect in drug therapy. The combined use of different types of cell death regulators (inducers or inhibitors) will be served as an alternatively therapeutic strategy. In some cases, some drugs have the capacity of simultaneously modulating different kinds of cell death demise. For instance, ketogenic diet was reported to suppress epileptic seizures by abrogating neuronal apoptosis and inducing autophagy (Wang et al., 2018). And the traditional medicine Curcumin evidently protected neurons against pilocarpine-induced status epilepticus in rats through inhibiting necroptosis and triggering autophagy (Wang et al., 2017b). Taken together, these data show that redox-related cell death modes including apoptosis, autophagy, necroptosis, pyroptosis, and ferroptosis are not independent. They are likely to interplay to form a network, ultimately mediating cell availability. Investigation of this network will definitely help us to better disclose the mechanism of seizure onset and/or the development of epilepsy.

## Concluding Remarks and Future Directions

Epilepsy is a highly complex neurological disorder, which is often accompanied with two features, namely, neuronal excitotoxicity and gliosis. On the one hand, neuronal hyperexcitation triggers ROS overproduction via enhancing calcium entry, finally leading to neuronal death. Neuronal ROS can also be transferred via neuron-glial gap junction, subsequently triggering neuroinflammation in glial cells (gliosis). On the other hand, following redox-related cell death, especially necroptosis, pyroptosis, and ferroptosis in neurons, the pro-inflammatory DAMPs are often released by the necrotic neurons and cause inflammatory responses in glial cells (gliosis). These two aspects both have an emphasis on the central roles of ROS and neuronal death mechanism in the development of epilepsy. Besides, we also give a proposal that ferroptosis, a recently identified regulated cell death mode, is involved in the etiology of epilepsy as we summarize the ferroptotic signs during epileptogenesis in Table 1. Ferroptosis and other types of redox-associated cell death including apoptosis, necroptosis, pyroptosis and autophagy may coexist within a neuron and these types of cell death modes can be interacted with each other in epilepsy. A deeper molecular understanding of ferroptosis and/or the interaction between different redox-neuronal death modes will undoubtedly lead to novel therapeutic approaches to mitigate epileptic seizures.

## Author Contributions

X-YM and W-LJ designed the manuscript. X-YM wrote the manuscript. W-LJ and H-HZ revised the manuscript.

## Conflict of Interest Statement

The authors declare that the research was conducted in the absence of any commercial or financial relationships that could be construed as a potential conflict of interest.

## Abbreviations

4-HNE: 4-hydroxy-2-nonenal protein adduct
AIF: apoptosis inducing factor
ALOX5: 5-lipoxygenase
Atg5: autophagy-related 5
DAMPs: damage-associated molecular patterns
ERK: extracellular regulated protein kinases
FOXO3: forkhead box O3
GABA_A_R: A type gamma-aminobutyric acid receptor
Gpx4: glutathione peroxidase 4
GSDMD: gasdermin D
JNK: c-Jun N-terminal kinase
KA: kainic acid
MAPKs: mitogen-activated protein kinases
MDA: malonaldehyde
MLKL: mixed lineage kinase domain-like
mPTP: mitochondrial permeability transition pore
NAC: *N*-acetyl-l-cysteine
NCOA4: nuclear receptor coactivator 4
NLRP3: NOD-like receptor pyrin domain-containing 3
NMDA: *N*-methyl-D-aspartate
PFT: pifithrin-a
RIPK3: receptor-interacting protein kinase 3
ROS: reactive oxygen species
TSC: tuberous sclerosis complex.
